# Stroke Awareness and Knowledge in the At-Risk Population: A Community-Based Study

**DOI:** 10.7759/cureus.57756

**Published:** 2024-04-07

**Authors:** Piandharm Dharmasaroja, Nattaphol Uransilp

**Affiliations:** 1 Senior High School, Triam Udom Suksa School, Bangkok, THA; 2 Division of Neurology, Department of Medicine, Thammasat University, Pathum Thani, THA

**Keywords:** ischemic stroke, be-fast, community-based study, vascular risk factor, stroke awareness

## Abstract

Background: The BE-FAST (balance, eyes, face, arms, speech, and time) mnemonic helps to identify stroke symptoms, which has been publicized through social media in Thailand for a while. Timely access to medical care enhances the likelihood of receiving efficacious treatment, thereby mitigating the adverse consequences of the stroke. Thus, stroke awareness in the general population, especially in the population at risk, is crucial. The purpose of the study was to evaluate stroke awareness and the level of knowledge about stroke in Thailand.

Methods: Adult participants aged at least 40 years, without a history of stroke, were included, with exclusions limited to those unwilling to participate. Data were gathered from the survey during two community visits and through an electronic survey via Google Forms (Google LLC, Mountain View, California, United States). A pre-tested questionnaire was divided into three parts: symptoms of acute stroke, vascular risk factors, and response. Demographics were compared between participants with good and poor knowledge of stroke awareness.

Results: A total of 281 participants were included, with a mean age of 58 years and 60% (n=169) of participants having at least one vascular risk factor. Of the responders, 133 (47%) had good knowledge of stroke awareness. Older age (age > 50 years, OR 0.326, 95%CI 0.173-0.615, p-value = 0.001), higher education (> 6 years, OR 0.266, 95%CI 0.139-0.508, p-value < 0.001)), and being female (OR 0.474, 95%CI 0.252-0.891, p-value = 0.020) were related to good stroke knowledge. Participants with good knowledge of stroke awareness also had good knowledge of vascular risk factors (84%; n=112), knew that they should come to the hospital immediately (74%; n=98), and also correctly identified the emergency calling number (90%; n=119).

Conclusions: Only half of the participants had good knowledge of stroke awareness. There is a need for improvement in the distribution of knowledge about stroke in Thailand.

## Introduction

The prevalence of stroke increases with the aging society [1]. The Thai Epidemiological Stroke (TES) study, which collected data from 19,997 subjects, aged 45-80 years, from five major geographical regions of the country, reported a prevalence of 1.88% [2]. The implementation of acute stroke interventions, including intravenous thrombolysis and mechanical thrombectomy, leads to lower morbidity and mortality in stroke patients [3,4]. One of the key factors that bring patients to receive acute stroke treatment is ‘Time’. The faster the application of acute treatment, the better outcomes are achieved [5]. Therefore, stroke awareness in the general population is important. Poor knowledge of stroke symptoms and risk factors has been shown to delay healthcare-seeking after a stroke attack [6,7]. Prehospital delay is also associated with face-to-face visits to the family doctor, while transportation by emergency medical services, severe stroke symptoms, and lower age reduces prehospital delay [7,8].

A study conducted on 217 Thai patients aged 29-92 years with acute stroke during 2010-2011 found that only one-third of patients were aware of stroke symptoms and 38% of patients came to the hospital within 4.5 hours [9]. Given the variations in study populations and methodologies across different countries and time periods, the level of awareness regarding stroke symptoms may vary. In 2018, a study in Egypt highlighted a poor level of stroke awareness among 1,154 individuals aged from under 25 years to more than 65 [10]. In 2021, research conducted in the Czech Republic involving 1,004 participants aged 18 years or older revealed a high overall level of stroke awareness [11]. Similarly, a study conducted in Riyadh, Saudi Arabia, in the same year, with 150 participants aged 18 to over 50 years, found that the majority (76%) exhibited a high level of awareness regarding stroke risk factors [12]. Additionally, over half of the participants (64%) showed moderate awareness about stroke signs and symptoms [12]. However, findings from a study in Jeddah, Saudi Arabia, indicated that although a majority of participants aged from under 20 years to older than 50 recognized the occurrence of stroke, a substantial portion (59%) were unable to correctly identify its symptoms [13].

The BE-FAST (Balance, Eyes, Face, Arms, Speech, and Time) algorithm helps to identify a person having an acute stroke [14,15]. The FAST mnemonic (Face, Arm, Speech, Time) has an 88% sensitivity for the identification of carotid distribution stroke but misses up to 40% of those with posterior circulation stroke [16]. Adding gait ataxia and visual symptoms leads to a reduction in missed strokes [15]. National Institute for Emergency Medicine and hospitals in Thailand have used BE-FAST to raise stroke awareness in the Thai population, which has been publicized through social media for a while. Social media can provide knowledge to people wider and faster than before. As the way of communication has changed and the importance of applying acute intervention to stroke patients in time has increased, it is necessary to learn how well the population at risk recognizes stroke awareness. The purpose of the study was to evaluate stroke awareness and the level of knowledge about stroke risk factors in the adult population and how quickly they should come to the emergency room (ER) in Thailand.

## Materials and methods

This was a cross-sectional, community-based study, conducted from October 2023 to January 2024. Adult participants aged at least 40 years old, without a history of stroke, were included, and exclusions were limited to those unwilling to participate in the survey. Data were gathered randomly from surveys during two community visits in Pathum Thani Province, specifically in the Chiang Rak and Klongluang districts, and through an electronic survey via Google Forms (Google LLC, Mountain View, California, United States), with the link being sent to interested participants. The research protocol was approved by the Human Ethics Committee of Thammasat University (project number: MTU-EC-IM-0-113/66).

A pre-tested questionnaire (see Appendices) was divided into three parts: symptoms of acute stroke (five correct answers; B (balance: gait ataxia/leg weakness), E (eyes: visual symptoms), F (facial droop), A (arm weakness), S (speech disturbance)), vascular risk factors (seven correct answers; hypertension, diabetes mellitus, hyperlipidemia, smoking, alcohol drinking, heart diseases), and how they should respond (time to come to ER after stroke onset and emergency calling number). They were multiple-choice questions with a few distractors on each question. The reliability of the questionnaire was assessed by three stroke experts with Cronbach’s coefficient alpha of 0.98. The content validity of the questionnaire was revised by two neurologists and one professional nurse with experience in stroke care and edited accordingly. The validity of the questionnaire was tested using the item-level content validity index (CVI), with an average CVI of 0.98. Responders who were able to choose three to five correct stroke symptoms were classified as having good stroke awareness. If they were able to identify at least three correct vascular risk factors (out of seven), they were classified as having good knowledge of vascular risk factors. The National Institute for Emergency Medicine has set a phone number (1669) to call for an ambulance in case of an emergency health problem, and stroke is one of them. In Thailand, the stroke network has been established to take care of or refer patients to receive appropriate acute stroke treatment. Two other questions were asked to assess how they responded in case of suspected acute stroke; one of them was the time within which the patient should seek medical treatment after symptom onset, and another was the emergency phone number.

The sample size was calculated using the formula:

 n = (P(1-P)Z^2^)/d^2^ ; (P=0.3, Z=1.65, d=0.1)

where n is the population size, P is the population proportion, Z is the z score, and d is the margin of error.

To account for potential dropouts or incomplete data, 10% of the calculated number was added. Thus, the sample size was calculated to be approximately 250. 

Information about the baseline characteristics of patients, including career and level of education, was collected. The data were presented as a mean or median for continuous variables and a percentage for dichotomous variables. The demographics were compared between participants with good and poor stroke awareness using the Student’s t test for the continued variables and the chi-square test for the dichotomous variables. Stepwise multivariate analyses were performed by including the pre-specified factors that were associated with good stroke awareness in the univariate analysis.

## Results

There were 281 adult participants in the study. Baseline characteristics are presented in Table 1. More than half of the responders (77%) were female, with a mean age of 58 years. One hundred and sixty-nine participants (60%) had at least one vascular risk factor.

**Table 1:** Baseline characteristics of subjects in the study (n=281).

Baseline Characteristics	Values
Mean age (years, range)	58 (40-85)
Female sex, n (%)	216 (77)
Median education (years, range)	14 (0-20)
≤ 6 years, n (%)	64 (23)
> 6 years, n (%)	217 (77)
Relatives having stroke history, n (%)	74 (26)
Hypertension, n (%)	100 (36)
Diabetes mellitus, n (%)	52 (19)
Hyperlipidemia, n (%)	93 (33)
Heart disease, n (%)	20 (7)
Smoking, n (%)	19 (7)
Numbers of vascular risk factors, n (%)	
0	112 (40)
1	99 (35)
2	42 (15)
3	18 (6)
4	4 (1)
5	6 (2)
Career, n (%)	
Company employee	44 (16)
Village health volunteer	41 (15)
Freelance career	32 (11)
Farmer	29 (10)
Merchant	25 (9)
Government employee	23 (8)
Public health officer	4 (1)
Housewife/ no career	55 (20)
Others	28 (10)

One hundred and thirty-three responders (47%) had good knowledge of stroke awareness. They were older, had a higher proportion of females, higher education, heart diseases, and a higher number of vascular risk factors compared to those with poor stroke awareness (Table 2). After adjusting for confounding factors, only older age (age >50 years; OR 0.326, 95%CI 0.173-0.615, p-value = 0.001), higher education (>6 years, OR 0.266, 95%CI 0.139-0.508, p-value < 0.001), and being female (OR 0.474, 95%CI 0.252-0.891, p-value = 0.02) were related to good stroke knowledge. Participants with good knowledge of stroke awareness also had good knowledge of vascular risk factors (84%) and knew that they should come to the hospital immediately (74%) and correctly identified the emergency calling number (90%) (Table 2).

**Table 2:** Associated factors of good stroke awareness. ^§^ Capable of choosing <3 correct stroke symptoms; ^§§^ Capable of choosing 3-5 correct stroke symptoms; * 95% confidence interval; ^**^ The p-value was calculated using Student’s t test for continuous variables and the chi-square test for dichotomous variables. Stepwise multivariate analyses were performed, incorporating pre-defined factors associated with good stroke awareness identified in the univariate analysis.

Baseline Characteristics	Poor stroke awareness ^§^ (n=148)	Good stroke awareness ^§§^ (n=133)	Odds ratio (95%CI) *	p-value **
Mean age (years)	56	60		0.023
< 50 years, n (%)	46 (31)	20 (15)		
≥ 50 years, n (%)	102 (69)	113 (85)	2.573 (1.427-4.640)	0.001
Female sex, n (%)	106 (72)	110 (83)	1.895 (1.067-3.365)	0.028
Mean education (years, range)	12	14		<0.001
≤ 6 years, n (%)	46 (31)	18 (14)		
> 6 years, n (%)	102 (69)	115 (87)	2.881 (1.571-5.285)	<0.001
Relatives having stroke history, n (%)	35, n=132 (27)	39, n=126 (31)	1.242 (0.724-2.133)	0.431
Hypertension, n (%)	51 (35)	50 (38)	1.857 (0.966-2.614)	0.514
Diabetes mellitus, n (%)	21 (14)	31 (23)	1.824 (0.988-3.364)	0.053
Hyperlipidemia, n (%)	42 (28)	51 (38)	1.570 (0.952-2.588)	0.076
Heart disease, n (%)	6 (4)	14 (11)	2.765 (1.030-7.418)	0.037
Smoking, n (%)	10 (7)	9 (7)	1.002 (0.394-2.545)	0.997
Numbers of vascular risk factors (mean, n)	0.87	1.16		0.034
0, n (%)	60 (41)	52 (39)		
1, n (%)	59 (40)	40 (30)		
2, n (%)	18 (12)	24 (18)		
3, n (%)	10 (7)	8 (6)		
4, n (%)	1 (1)	3 (2)	1.361 (1.006-1.843)	0.042
5, n (%)	0	6 (5)		
Numbers of vascular risk factors, n (%)				
0-1	119 (80)	92 (69)		
≥ 2	29 (20)	41 (31)	1.829 (1.057-3.163)	0.030
Good knowledge, n (%)				
Knows vascular risk factors	32 (22)	112 (84)	19.333 (10.520-35.532)	<0.001
Need to reach ER immediately	84 (57)	98 (74)	7.817 (5.622-14.017)	<0.001
Knows emergency calling number	83 (57)	119 (90)	1.477 (1.193-4.827)	<0.001

For stroke symptoms, BE-FAST has been publicized in Thailand for a while. Balance problems were correctly identified as one of the stroke symptoms by 117 participants (42%). Others were Eyes: 110 (39%), Facial droop: 177 (63%), Arm weakness: 181 (64%) and Speech: 153 (54%). For the question about how fast they should come to the ER if they suspected an acute stroke, 182 responders (65%) answered ‘immediately’; others stated within 4.5 hours (n=43; 15%), within 6 hours (n=3; 1%), within 24 hours (n=18; 6%), within three days (n=1; 0.4%), and did not know (n=34; 12%).

## Discussion

The advances in acute ischemic stroke treatment, including treatments aiming to recanalize and reperfuse the ischemic brain, lead to lower morbidity and mortality in patients [3,4]. The time from stroke onset to accessing treatment is crucial, especially for those who have large vessel occlusion. A 30-minute delay in recanalization increases the risk of moderate or severe disability by 7% and the risk of death by 11.8% [17]. One of the risk factors of prehospital delay is a lack of awareness of stroke symptoms, and another is face-to-face visits to family doctors [7]. Severe strokes, the use of ambulances, and lower age are associated with reduced prehospital delay, and more than half of the delay is caused by the hesitation to contact medical services [8]. Thus, knowledge about stroke awareness and immediate response in the general population is important.

The older age of participants and having vascular risk factors seem to be the factors that encourage individuals to learn about stroke symptoms. Based on the Thailand epidemiological study, which collected data from 19,997 subjects aged 45-80 years from five major geographical regions of the country, the stroke prevalence is 1.88% [2]. Our present study aimed to evaluate stroke awareness knowledge in the adult population; thus, we included participants aged at least 40 years without a history of stroke. Abdalla et al. reported that 26.6% of the studied population in Riyadh, Saudi Arabia, had good knowledge about stroke, which included stroke symptoms, and the older population (aged at least 45 years) had the highest proportion of good knowledge [6]. Another study of 314 patients with diabetes mellitus revealed that 69% of the patients had a good level of knowledge of stroke risk factors and symptoms [18]. Our study showed that almost half of the participants had good knowledge of stroke symptoms, which was associated with older age, higher education, and female gender. The mean age of participants in our study was 58 years and 60% of participants had at least one vascular risk factor, which was the primary target of the study because this group was the population at risk of developing a stroke. The elderly population may, in part, have experience of their close friends or relatives having a stroke and those who have vascular risk factors would have regular appointments with doctors, where they can be educated. This would encourage them to learn about stroke symptoms and how they should respond if they have stroke symptoms. In Thailand, subdistrict administrative organization regularly provides health education to the community, especially through village health volunteers. Women usually have more social engagement than men and more than half of village health volunteers are women [19]. Thus, women tend to have higher knowledge about stroke awareness than men.

Symptoms that cause disability, such as arm weakness, may prompt patients to seek earlier medical attention. Arm weakness (64%) was the most commonly identified symptom of stroke by participants in our study. Others were facial droop (63%), speech impairment (54%), gait ataxia (42%) and visual symptoms (39%). Other studies, one conducted in a hospital-based setting in Norway and the other involving diabetic patients in Saudi Arabia, also showed that weakness on one side of the body and difficulty in speaking or more severe stroke symptoms were the most common signs detected by responders [8,18].

Social media-based campaigns using advertisements provide feasibility and cost-effectiveness to raise stroke awareness in the general population [20]. Such campaigns can be particularly effective during pandemic disasters like the coronavirus disease 2019 pandemic, with lower costs compared to in-person or paper distribution methods [21]. Our study revealed a higher proportion (47%) having good stroke awareness compared to a previous study of Thai patients during 2010-2011, which found that only one-third of patients with a stroke were aware of stroke symptoms [9]. However, the government should continue to publicize periodically to raise stroke awareness and promote healthcare concerns to prevent stroke in the general population. Promoting the utilization of social media, especially in developing countries, is crucial for disseminating dependable information and fostering global user engagement [22].

Although the participants in our study were the population at risk of developing stroke, there were a few limitations in our study. First, the number of studied participants may be too small to represent the whole population. Second, most participants lived in Pathum Thani, which is a Bangkok suburb, and had a mean education of 13 years. This may not be representative of the population in the countryside. Further studies with a larger sample size from a variety of regions may be needed.

## Conclusions

Only half of the participants demonstrated good stroke awareness, with older age, higher education, and female gender associated with better knowledge. Our findings underscore the importance of targeted interventions aimed at improving stroke awareness in Thailand, particularly among demographic groups identified as having lower levels of knowledge, such as younger individuals, those with lower education levels, and males. This could be achieved by implementing targeted campaigns through various channels such as digital platforms or tailoring educational interventions to specific demographic groups. Stroke symptom awareness among the general population can impact stroke management by enabling earlier recognition and presentation of stroke cases to healthcare facilities, ultimately improving outcomes and reducing the burden of stroke-related disabilities. Additionally, increased awareness may encourage individuals to adopt preventive measures and seek medical attention promptly.

## Appendices

Questionnaire

NOTE: The English version of this questionnaire has been translated from Thai.

General information

1. From where did you learn about this survey?

o   Links from social media platforms like LINE.

o   From outreach or community engagement activities of public health agencies.

2. Gender

o   Male

o   Female

3. Age (years)

4. Education level

o   Doctorate or equivalent

o   Master's degree or equivalent

o   Bachelor's degree or equivalent

o   Professional certificate at the advanced level

o   High school diploma or vocational certificate

o   Middle school

o   Elementary school

o   No formal education

5. Occupation         

o   Health official/hospital staff

o   Volunteer in community health service

o   Government employee

o   Trader

o   Company employee

o   Freelancer

o   Farmer/Gardener

o   Homemaker/Unemployed

o   Other

6. Do you know an acquaintance or have a relative who has had a stroke?

o   Yes

o   No

7. Do you have any existing medical conditions or risk factors for cardiovascular disease? (You can select more than one option)

o   High blood pressure

o   High cholesterol

o   Diabetes

o   Heart disease

o   Smoking

o   None

o   Other

Knowledge about stroke

1. How many types of strokes are there?

o   1 type (Ischemic stroke OR Hemorrhagic stroke)

o   2 types (Ischemic stroke AND Hemorrhagic stroke)

o   Not sure / Don't know

2. Common symptoms of stroke include: (You can select more than one option)

o   Sudden facial drooping or asymmetry

o   Headache

o   Fever

o   Difficulty speaking or understanding speech

o   Dizziness, vertigo, loss of balance

o   Weakness or paralysis in arms or legs

o   Not sure / Don't know

3. Causes or risk factors commonly associated with stroke include: (You can select more than one option)

o   High blood pressure

o   Insomnia

o   Stress

o   Diabetes

o   High cholesterol

o   Smoking

o   Alcohol consumption

o   Heart disease

o   Obesity

o   Not sure / Don't know

4. If you suspect symptoms of stroke, when should you see a doctor to receive prompt treatment?

o   Immediately

o   Within 3 or 4.5 hours

o   Within 6 hours

o   Within 24 hours

o   Within 3 days

o   Not sure / Don't know

5. Emergency phone number in case of suspected stroke symptoms and unable to help oneself:

o   191

o   1669

o   1668

o   Not sure / Don't know
